# Does student performance on preclinical OSCEs relate to clerkship grades?

**DOI:** 10.3402/meo.v21.31724

**Published:** 2016-06-22

**Authors:** Margot Chima, Gary Beck Dallaghan

**Affiliations:** Curriculum and Educational Research Office, The University of Nebraska Medical Center, Omaha, NE, USA

**Keywords:** clinical education, communication skills, curriculum development

## Abstract

**Background:**

Objective structured clinical examinations (OSCEs) have been used to assess the clinical competence and interpersonal skills of healthcare professional students for decades. However, the relationship between preclinical (second year or M2) OSCE grades and clerkship performance had never been evaluated, until it was explored to provide information to educators at the University of Nebraska Medical Center (UNMC). In addition, the relationship between M2 OSCE communication scores (which is a portion of the total score) and third-year (M3) Internal Medicine (IM) clerkship OSCE scores was also explored. Lastly, conflicting evidence exists about the relationship between the amount of previous clinical experience and OSCE performance. Therefore, the relationship between M3 IM clerkship OSCE scores and the timing of the clerkship in the academic year was explored.

**Methods:**

Data from UNMC M2 OSCEs and M3 IM clerkship OSCEs were obtained for graduates of the 2013 and 2014 classes. Specifically, the following data points were collected: M2 fall OSCE total, M2 fall OSCE communication; M2 spring OSCE total, M2 spring OSCE communication; and M3 IM clerkship OSCE total percentages. Data were organized by class, M3 IM clerkship OSCE performance, and timing of the clerkship. Microsoft Excel and SPSS were used for data organization and analysis.

**Results:**

Of the 245 records, 229 (93.5%) had data points for all metrics of interest. Significant differences between the classes of 2013 and 2014 existed for average M2 spring total, M2 spring communication, and M3 IM clerkship OSCEs. Retrospectively, there were no differences in M2 OSCE performances based on how students scored on the M3 IM clerkship OSCE. M3 IM clerkship OSCE performance improved for those students who completed the clerkship last in the academic year.

**Conclusions:**

There were inconsistencies in OSCE performances between the classes of 2013 and 2014, but more information is needed to determine if this is because of testing variability or heterogeneity from class to class. Although there were no differences in preclinical scores based on M3 IM clerkship OSCE scores, students would benefit from a longitudinal review of their OSCE performance over their medical training. Additionally, students may benefit from more reliable and valid forms of assessing communication. In general, students who take the IM clerkship last in the academic year performed better on the required OSCE. More information is needed to determine why this is seen only at the end of the year.

Objective structured clinical examinations (OSCEs) have been used to assess the clinical competence and interpersonal skills of healthcare professional students for decades (1). The reliability and utility of these examinations has been widely studied (2) and the correlation between OSCE performance and many other metrics has been described. These metrics include performance on future OSCEs; residency performance (3); NBME subject exams; USMLE Steps 1, 2CK, 2CS, and 3 (4, 5); and medical school grade point averages (6).

No published accounts of the relationship between medical student performance on preclinical OSCEs and clerkship grades were identified. This novel association could be important in understanding if students are consistently good/poor performers on OSCEs through medical school. This has implications for medical schools to achieve minimal competence with entrustable professional activities related to patient communications (7, 8). Students should demonstrate improved skills with each OSCE if the competency-based approach to medical education is accurate.

Additionally, the reliability of OSCEs to effectively assess student performance varies based on many factors, including number of stations during the examination, the type of examiner (standardized patient, senior student, or faculty), how heavily various skills are weighted in calculating the total grade (communication, clinical reasoning, or charting), and the type of rating scale (checklist or Likert scale) (2). Furthermore, it is harder to reliably assess communication skills than procedural or clinical skills with this type of examination (2).

Although difficult to measure, medical educators need to be able to reliably assess communication. In a 2014 survey of important factors in ranking applicants, residency program directors valued interpersonal skills with the highest reported rating of importance. Interpersonal skills were cited as being more important in influencing residency decisions than grades and standardized test scores (9). This reflects the influence of competency-based education in residency training (10). Therefore, objectifying, stratifying, and improving students’ ability to communicate is imperative during medical school education.

Turner et al. describes the gravity of the situation.

Successful OSCEs are often the result of significant planning, coordination of multiple resources, commitment to large-scale testing, and judicious use of assessment data. Care must be taken to minimize the multiple sources of error and find validity evidence to justify OSCE use. Such attention to these issues -to do it right- comes with a hefty price tag. When high-stakes consequences hang in the balance, however, it is essential that these details are not taken lightly. (11)

This is no exception at the University of Nebraska Medical Center (UNMC). Many resources go into planning and administering preclinical second-year (M2) medical student OSCEs, and it is unknown if there is a correlation with third-year (M3) Internal Medicine (IM) clerkship OSCE performance. Knowing this could help medical educators identify students who require intervention, particularly before residency interviews.

Additionally, conflicting data exist on the relationship between clinical exposure and student performance on OSCEs (12–14). For this reason, the relationship between M3 IM clerkship OSCE scores and the timing of the clerkship (first, second, third, or fourth) in the academic year was assessed, presuming clinical exposure increases as the year progresses. We assessed these relationships in order to provide valuable information for medical educators that will hopefully serve as a springboard for further investigation.

## Hypotheses

In order to understand the relationship between preclinical and clerkship OSCE performances, the following hypotheses were tested:

1. H_0_: There is no difference in preclinical OSCE performance based on clinical OSCE scores.
  H_1_: There is a difference in preclinical OSCE performance based on clinical OSCE scores.
2. H_0_: There is no difference in preclinical OSCE communication scores based on clinical OSCE scores.
  H_1_: There is no difference in preclinical OSCE communication scores based on clinical OSCE scores.
3. H_0_: There is no difference in the relationship of clinical OSCE scores based on timing of the internal medicine clerkship.
  H_1_: There is a difference in the relationship of clinical OSCE scores based on timing of the internal medicine clerkship.

## Methods

Data from the UNMC medical student M2 OSCEs and M3 IM clerkship OSCEs were obtained for graduates of the 2013 and 2014 classes. Specifically, the following percentages for each student were collected: M2 fall OSCE total, M2 fall OSCE communication; M2 spring OSCE total, M2 spring OSCE communication; and M3 IM clerkship OSCE total (note: the terms *score* and *percentage* will be used interchangeably henceforth). Only students with all data points mentioned, collected between 2011 and 2013, were included in the analysis. The data were analyzed using Microsoft Excel and SPSS version 23.

Variables were analyzed for normal distribution. Shapiro–Wilk tests confirmed that none of the variables, with the exception of the M2 spring OSCE total, were normally distributed. Therefore, only non-parametric tests were used in analysis.

All scores for the classes of 2013 and 2014 were compared using Mann–Whitney U tests to determine any differences in scores between the two classes.

In order to determine if a correlation existed between M3 IM clerkship OSCE performance and any of the M2 OSCE scores, Spearman's rho correlation tests were used. Spearman's rho correlation analysis was also used to assess the correlation between preclinical M2 OSCE communication and total percentages. Before performing these analyses, scatter plots demonstrated monotonicity between the assessed variables.

For administrative reasons, the data were then stratified into subgroups based on student performance on the M3 IM clerkship OSCE. These groups were defined by total score in the 90s (90–99%), 80s (80–89%), 70s (70–79%), and 60s (60–69%). Kruskal–Wallis tests were used to determine if significant differences in performance on any of the M2 OSCE scores existed between the subgroups created by the M3 IM clerkship OSCE score stratification.

In order to determine if average M3 IM clerkship OSCE scores differed based on when students rotated through the IM clerkship in the academic year, the data were categorized by clerkship timing (first, second, third, or fourth). A Kruskal–Wallis test was performed to assess the differences in OSCE performance between these groups.

## Results

Of the 245 records, 229 (93.5%) had data points for all the metrics of interest. Incomplete records came from students who did not progress in their education, which may have included students withdrawing or dual degree students (e.g., MD/PhD). Of those students analyzed, 116 (50.66%) were represented in the class of 2013 and 113 (49.34%) in the class of 2014.

Mann–Whitney U tests revealed significantly higher M2 spring OSCE total and communication scores for the class of 2013; M3 IM clerkship OSCE scores were higher for the class of 2014 (Table 1). For all M2 OSCEs, median communication scores always exceeded total scores (Table 1).

**Table 1:** Mann–Whitney U results for the class of 2013 versus the class of 2014

	N	Mean (%)	Median (%)	Range (%)	U	*p*
M2 fall OSCE total						
Class of 2013	116	90.31	90.96	79.69–99.38	5,851	0.161
Class of 2014	113	89.06	90.29	67.96–97.20		
Total	229	89.69	90.80	67.96–99.83		
M2 fall OSCE communication						
Class of 2013	116	95.73	100.00	60.87–100.00	6,518	0.936
Class of 2014	113	96.15	100.00	69.57–100.00		
Total	229	95.94	100.00	60.87–100.00		
M2 spring OSCE total						
Class of 2013	116	86.27	86.12	72.46–97.55	3,234	0.000*
Class of 2014	113	81.34	81.67	68.33–91.67		
Total	229	83.83	84.17	68.33–97.55		
M2 spring OSCE communication						
Class of 2013	116	96.78	100.00	82.61–100.00	5,430	0.016*
Class of 2014	113	94.34	95.65	60.87–100.00		
Total	229	95.58	95.65	60.87–100.00		
M3 IM clerkship total						
Class of 2013	116	83.53	84.44	68.13–94.29	7,651	0.029*
Class of 2014	113	84.95	85.63	82.25–93.81		
Total	229	84.23	85.00	68.13–94.29		

* Statistically significant.

M3 IM clerkship OSCE performance was very weakly correlated with each of the M2 OSCE scores, but these correlations were not significant (Table 2). However, when only the preclinical data were analyzed, there was a significant (weakly) positive correlation between M2 communication and total scores. This was observed for both the fall (*r*_s_=0.29, *p*<0.01) and spring (*r*_s_=0.27, *p*<0.01) OSCEs.

**Table 2:** Spearman's Rho results for M3 IM clerkship OSCE scores and M2 OSCE scores

	M2 fall OSCE total	M2 fall OSCE communication	M2 spring OSCE total	M2 spring OSCE communication
Chi-square	0.967	1.574	0.774	0.353
df	2	2	2	2
Asymp. Sig	0.617	0.455	0.679	0.838

The stratification of data into groups by percentages yielded 23 (10.04%) scoring in the 90s, 166 (72.49%) scoring in the 80s, 39 (17.03%) scoring in the 70s, and 1 (0.44%) scoring in the 60s. As Kruskal–Wallis testing cannot assess variance for a group when *n*=1, the data point for the one student scoring in the 60s was excluded from analysis. No matter how students performed on the M3 IM clerkship OSCE (90s, 80s, 70s), there were no differences in preclinical OSCE performances between the groups (Table 3).

**Table 3:** Kruskal–Wallis results for M3 IM clerkship OSCE scores (90s, 80s, and 70s)

	Between M3 IM clerkship OSCE and			
	
	M2 fall OSCE total	M2 fall OSCE communication	M2 spring OSCE total	M2 spring OSCE communication
*r_s_*	0.10	0.11	0.06	−0.07
*p*	0.12	0.10	0.34	0.27

When M3 IM clerkship data were stratified by timing of the clerkship, there were no differences in performance whether students completed the clerkship first, second, or third in the academic year. Students who completed their internal medicine clerkship last, did significantly better (H (2)=16.407, *p*<0.01) than all other times during the academic year (Table 4).

**Table 4:** Mean M3 IM clerkship OSCE scores by timing

	First	Second	Third	Fourth	Total
Mean (%)	83.47	83.82	84.25	86.78*	84.78
N	60	60	63	46	229

* Statistically significant.

## Conclusions

Average M2 spring total, M2 spring communication, and M3 IM clerkship OSCE scores differed between the classes of 2013 and 2014, which could reflect inconsistencies in administration and grading of each test year-to-year. This could also reflect class-to-class heterogeneity of OSCE ability, but more data are needed. If the decision is made to change the grading or administering of OSCEs in the future, continued analysis year-to-year will be important to assess future testing consistency.

Overall student performance during M2 fall and spring OSCEs demonstrated no association with M3 IM clerkship OSCEs; therefore, null hypothesis 1 cannot be rejected. This could possibly be explained by differences in the administration and grading of M2 versus M3 OSCEs. Standardization of stations and checklists between preclinical and clinical OSCEs could provide better information for understanding student performance over time. Although there are very weak correlations between M2 OSCE grades and M3 IM clerkship OSCE performance, small sample size most likely contributed to the insignificance of these relationships.

Looking back, no matter how students performed on the M3 IM clerkship OSCE (90s, 80s, 79s), there were no differences in their previous preclinical communication performance during the M2 year. Therefore, null hypothesis 2 cannot be rejected. During M2 OSCEs, the median communication portion always exceeded the average total scores, which could reflect that the current method of grading communication fails to effectively stratify students (too many high scores).

There was a weakly positive correlation between preclinical OSCE communication and total scores. This could suggest that communication scores, although contributing somewhat, are still underrepresented in the calculation of the total score. Because interpersonal and communication skills are imperative yet difficult to measure, more effective means of assessing student communication should be instituted at UNMC. For example, the MAAS-Global rating list for doctor–patient communication skills has been shown to be relatively more valid and reliable than other means of assessing student communication similar to the method used during this study (15, 16). Because only a weak correlation existed between preclinical communication and total OSCE scores, communication should be weighted more heavily in the total OSCE percentage to reflect its importance.

In general, M3 IM clerkship OSCE performance does not improve as the academic year progresses, with the exception of the last group of students to rotate through the clerkship. Therefore, null hypothesis 3 can be rejected. More information is needed to examine why student performance only increases for students taking their internal medicine clerkship last, versus steadily increasing as the year progresses.

Furthermore, all students may benefit from a longitudinal review of their OSCE communication scores and comments. In conversation with UNMC Assistant Dean for Medical Education and Director of the Office of Medical Education, Gary L. Beck Dallaghan, PhD (November 2015), this will be a part of the curriculum redesign as means of reaching milestones related to this entrustable professional activity.
